# Pain Reconceptualisation after Pain Neurophysiology Education in Adults with Chronic Low Back Pain: A Qualitative Study

**DOI:** 10.1155/2018/3745651

**Published:** 2018-09-12

**Authors:** Richard King, Victoria Robinson, Helene L. Elliott-Button, James A. Watson, Cormac G. Ryan, Denis J. Martin

**Affiliations:** ^1^The Pain Clinic, South Tees Hospitals NHS Foundation Trust, Middlesbrough TS3 4BW, UK; ^2^Health and Social Care Institute, Teesside University, Middlesbrough TS1 3BA, UK

## Abstract

Pain neurophysiology education (PNE) is an educational intervention for patients with chronic pain. PNE purports to assist patients to reconceptualise their pain away from the biomedical model towards a more biopsychosocial understanding by explaining pain biology. This study aimed to explore the extent, and nature, of patients' reconceptualisation of their chronic low back pain (CLBP) following PNE. Eleven adults with CLBP underwent semistructured interviews before and three weeks after receiving PNE. Interviews were transcribed verbatim and thematically analysed in a framework approach using four a priori themes identified from our previous research: (1) degrees of reconceptualisation, (2) personal relevance, (3) importance of prior beliefs, and (4) perceived benefit of PNE. We observed varying degrees of reconceptualisation from zero to almost complete, with most participants showing partial reconceptualisation. Personal relevance of the information to participants and their prior beliefs were associated with the degree of benefit they perceived from PNE. Where benefits were found, they manifested as improved understanding, coping, and function. Findings map closely to our previous studies in more disparate chronic pain groups. The phenomenon of reconceptualisation is applicable to CLBP and the sufficiency of the themes from our previous studies increases confidence in the certainty of the findings.

## 1. Introduction

Pain neurophysiology education (PNE) has become a commonly used educational intervention for patients with chronic pain. PNE is a cognitive behavioural-based intervention in that it aims to reduce inappropriate beliefs and maladaptive behaviours, in order to decrease pain and disability, by explaining the biology of pain to the patient [1]. A growing body of literature supports its effectiveness [2–10].

Patients with chronic pain, fuelled by health care professionals, often hold strong biomedical model beliefs that their pain is due to tissue damage [11–14]. A number of conceptual models have proposed that such inappropriate beliefs can lead to the development/maintenance of chronic pain. Within the fear-avoidance model, when pain is perceived as threatening, catastrophic thinking can result in pain-related fear and anxiety, leading to avoidance behaviour, disability, and a vicious cycle of chronic pain [15]. Additionally, as proposed within the model of misdirected problem-solving, inappropriate beliefs about tissue damage housed within a medical model framework can lead patients with chronic pain to repetitively seek solutions to remove their pain, moving from one treatment to the next, stuck within a *perseverance loop*. Each unsuccessful solution amplifies the condition and can prevent the patient from reframing their efforts away from an arguably unachievable goal of pain cessation to one of pursuing a *valued life in the presence of pain* [16].

A primary mechanism by which PNE purports to work is by helping patients better understand their pain and issues around its causes, correcting inappropriate beliefs—reconceptualising their pain [17]. Reconceptualisation can be defined by four key concepts: *(i) pain does not provide a measure of the state of the tissues; (ii) pain is modulated by many factors across somatic, psychological, and social domains; (iii) the relationship between pain and tissue becomes less predictable as pain persists; and (iv) pain can be conceptualised as a conscious correlate of the implicit perception that tissue is in danger* [17]. In theory, pain reconceptualisation should reduce the commonly perceived fear that pain is a clear signal of tissue damage by dispelling the notion that pain is an accurate indication of the state of tissue. Reduction of this fear may lead to reduced pain-related fear, distress, and disability; improved physical and mental health [15, 18]; an escape from the perseverance loop identified within the misdirected problem-solving model [16]; and potentially reduced levels of pain [8].

Only a few studies have been carried out exploring the phenomenon of reconceptualisation as a key mechanism of PNE. Evidence that PNE improves participants' knowledge of pain neurophysiology and reduces fear avoidance and pain catastrophising has been used to imply that reconceptualisation is a key factor [3, 4, 19, 20]. However, the narrow scope of the outcome measures (using structured questionnaires) in these studies provides limited insight into the complex phenomenon of pain reconceptualisation, and a validated questionnaire for the measurement of reconceptualisation has not been developed. At this stage of the development of evidence, qualitative methodology is better suited to studying pain reconceptualisation as it allows for an indepth exploration of multifaceted phenomenon [21] such as reconceptualisation. Our previous studies have found that patients with chronic pain often hold conflicting views about the cause/nature of their pain. Qualitative methods can help to reveal and explore these conflicting complex beliefs to an extent that quantitative methods cannot [22].

Two recent qualitative studies completed by our group identified the level of pain reconceptualisation following a single 2-hour session of PNE in patients with chronic pain as “partial and patchy” [23, 24]. However, where degrees of reconceptualisation were evident, we also saw clinical improvements, supporting the idea that reconceptualisation is a central mechanism of PNE's effect. A notable finding was the importance of relevance of PNE to the individual's specific experience as opposed to being relevant to a more general experience of living with pain [23, 24]. The participants included in these two studies were from a range of pain conditions including multisite pain, lower back pain (with and without leg pain), thoracic pain, throat pain, complex regional pain syndrome, neck pain, and upper limb pain. A key factor which may impact upon relevance to the patient is their pain condition and how they perceive PNE fits with their symptoms. Poor perceived fit between symptoms and PNE may reduce perceived relevance for the patient. “*For me personally I didn't think it was any good for the symptoms that I have… it was for more for people with different parts of the body pain and not the one I have*” [24]. Thus, looking at the experience of PNE for specific pain populations may be important.

In Robinson et al. [23], four participants out of a total of 10 demonstrated some evidence of reconceptualisation following PNE. All four had multisite pain. In contrast, two of the four participants with chronic low back pain (CLBP) reported that PNE was not relevant to them, they perceived no benefit, and showed no signs of reconceptualisation. Within educational theory, conceptual change requires a dissatisfaction with one's current understanding of a concept [25]. For many, perhaps most people, there is a strong belief that back pain can be readily aligned with the medical/tissue injury model [26]. This gives rise to the possibility that they may be more accepting of a biomedical explanation and thus less open to reconceptualisation than people with multisite pain or painful conditions that defy the logic of a medical model explanation. It may also be that they are less likely to have encountered an alternative explanation for their pain beyond the medical model. This corresponds with observations we made from previous work [24], where a participant with CRPS, a condition that fits poorly with the medical model, demonstrated pain reconceptualisation following PNE and showed clear signs of an awareness and understanding of pain hypersensitivity before receiving PNE.

Chronic low back pain (CLBP) is a particularly important pain subgroup to focus upon as it is one of the most common pain conditions globally and it is the largest single cause of disability-adjusted life years (2,313 per 100,000 population) in the UK [27]. The National Institute for Health and Care Excellence (NICE) estimates that back pain costs the UK economy over 2.1 billion annually [28]. Considering the potential importance of the person's pain condition with respect to perceived relevance, reconceptualisation, and ultimately the effectiveness of PNE, there is a need to explore pain reconceptualisation in people with CLBP following PNE. In doing so, new approaches to tailoring and enhancing this education specifically for patients with CLBP may be identified. Thus, the aim of this study was to investigate the extent, and nature, of people's reconceptualisation of their CLBP following PNE.

## 2. Materials and Methods

### 2.1. Design

We used the approach of theoretical thematic analysis [29] with a focus towards deductive analysis to explore the applicability of the themes we had found in our previous work on people with chronic pain in general [23, 24] to a group with CLBP only. Due to the heterogeneity of this study sample, we felt that it was important to be open to exploring the data for any additional/new themes that may emerge. To reflect this, we also used inductive analysis.

### 2.2. Recruitment and Sample

Participants were recruited from a single site—an NHS pain clinic in the North East of England. We aimed to recruit a convenience sample of 10–12 participants. While no formal guidelines exist with respect to sample size estimation for qualitative studies, it has been proposed that in studies where the aim is to understand common perceptions and experiences, twelve interviews should be sufficient [30]. Patients were eligible for inclusion if they had been referred to PNE as part of their usual care, were ≥18 years of age, and if their primary complaint was chronic (>6 months duration) lower back pain (±leg symptoms) of a neuro/musculoskeletal origin. All referrals were made by consultants in pain management following assessment. None of the participants required spinal or orthopaedic surgery.

Patients were excluded from the study if their level of English was not judged suitable enough to take part in an interview or if their pain was not primarily associated with the musculoskeletal system such as neurological conditions. To limit any feeling of coercion, patients of the interviewer (RK) were also excluded from taking part in the study. Patients with the primary complaint of LBP who had been referred to PNE as part of their usual care were sent a brief information sheet regarding the study. Following this, the patient was contacted by a research assistant and asked if they would like to receive more information regarding the study. If they did, this information was sent to them and they were contacted to see if they would like to participate. Data were collected between September and November 2014. This study was approved by NRES Committee Yorkshire and The Humber – Sheffield (REC Reference number: 14/YH/0153). Written informed consent was obtained from all participants before they entered the study. On completion of data collection, all data were fully anonymised.

### 2.3. Intervention

All participants in this study received PNE as part of their routine usual NHS care. The PNE session was heavily based upon the manual *Explain pain* [1]. The PNE session was delivered in a group setting of 10–12 patients with chronic pain. The patients within the groups were heterogeneous with respect to their clinical condition; however, only people with CLBP were recruited into this study. Thus, the PNE delivered was not back pain specific. The intervention was delivered by two experienced, pain specialist physiotherapists who have worked within the pain setting for >5 years each, had undertaken postgraduate training in pain, and attended *Explain Pain* courses delivered by the Neuro Orthopaedic Institute. Published service evaluation data have shown that patients with chronic pain who receive PNE at this clinic demonstrate average increases in pain knowledge in keeping with increases reported in the literature [31, 32].

### 2.4. Data Collection

Participants underwent a semistructured interview one week prior to PNE. The interview script is provided in Supplementary Material (available here). The pre-PNE interview focused on beliefs about the nature, cause, and experiences of their pain. Three weeks after PNE, participants were reinterviewed by the same researcher using the same semi-structured approach. Participants were asked the same questions as in the first interview but were also asked to reflect on any change in their understanding of their pain. All interviews took place in the hospital in a private room lasting approximately one hour, with only the interviewer and participant present. They were audio recorded and transcribed verbatim for thematic analysis.

### 2.5. Analysis

The primary analysis of the data was conducted by RK using NVivo software (version 10), following the guidelines for theoretical thematic analysis outlined by Braun and Clarke [29]. Each transcript was read multiple times and statements were coded according to their meaning. Coded statements were grouped together into four a priori themes that we found in our previous work [23, 24]—degrees of reconceptualisation, personal relevance, importance of prior beliefs, and perceived benefit of PNE. We also provided for the emergence of themes that did not fit with the above.

To ensure dependability, all views were treated equally. Three weeks following the second interview, RK telephoned all participants to verify that he had an accurate interpretation of the participants account. Only 8 participants could be contacted. During the telephone conversation, extracts from the interview were described to the participant to assess/verify if the researcher had made an appropriate interpretation of the interview comments. In all cases, the participants agreed with the interpretation of the account. Therefore, no amendments were made. The average duration of the telephone conversation was 12 minutes. Following this process, a second researcher (HE) read all the transcripts to ensure the themes were logical and rooted in the data. To increase credibility, the results were circulated throughout the rest of the research team for further refinement and to be collected into a coherent account.

Evidence for or against the a priori themes was sought from participants' subjective accounts and changes were explored by comparing participants' pre- and post-PNE interviews.

### 2.6. Reflexivity

Reflexivity relates to the amount of influence the researcher—consciously or unconsciously—has on the outcome of the study and can be defined as “*a continuous process of reflection by the researcher on their values, preconceptions, behaviour, or presence and those of the participant which can affect interpretation of responses*” [33]. Therefore, disclosure of the researchers' standpoints allows the reader to consider how this might have impacted on the findings. To this end, four of the researchers (RK, VR, JW, and CR) have experience of delivering PNE. RK and VR have extensive experience in pain management (6 and 11 years' full-time physiotherapists in pain management, resp.), regularly deliver PNE as part of their clinical practice, and have undertaken professional training to do so. It is their (RK, VR, JW, and CR) belief that PNE is a clinically useful intervention; however, they have no vested interest in the outcome of this study. DM and HE do not have experience of delivering PNE clinically. Their involvement is from a research method's perspective. They support the potential underlying theory of reconceptualisation and remain open to the theories being shaped by evidence.

## 3. Results

Out of 12 participants initially recruited, only 11 provided a pre- and postinterview. One participant did not provide a postinterview (Participant 6). This individual did not supply a reason for this and we did not have ethical approval to approach her to find out why she did not attend (Table 1). Of the 12 participants, 7 were female and 5 were male. All participants were diagnosed with low back pain of greater than 6 months duration. The average (range) duration of pain was 10 years and 4 months (8 months–26 years). The average (range) age of participants was 48 years (25–72 years). Of the 12 participants, 3 were unemployed, 6 were employed, and 3 were retired. Participants ranged from having no qualifications to holding a BSc (Hons) degree. A summary of how each participant was analysed against the a priori themes is shown in Table 1. Additional themes, beyond those identified a priori, did not emerge from the data.

### 3.1. Theme 1: Degrees of Reconceptualisation

No evidence for reconceptualisation was found in the accounts of Participants 9, 11, and 12. Following PNE, their explanations of the current cause of their pain were expressed exclusively in biomedical language, as was the case before PNE.

*“When they done the MRI, when they done that, they discovered I had this impingement in my spine.”* (P9 pre)

*“The reason why I'm in pain? Because of my impingement...”* (P9 post)

We observed evidence of reconceptualisation in the accounts of P1, 2, 3, 4, 7, 8, and 10. This evidence took various forms: language that no longer discussed pain in purely biomedical terms, the use of neurophysiological terms in a way that was not evident in the interviews before PNE, new language about the links between pain and emotions.

P10's shift from an entirely biomedical view of her pain to becoming open to the idea that such an explanation may not be sufficient is illustrative.

*“…I won't have that made as an excuse for this because there's something real happening in my back. I think there's something wrong with my discs.”* (P10 pre)

*“…there might not be [a structural] explanation for it…as it was explained in the session last week, it might not be structural.”* (P10 post)

For P1, 2, 3, 7, and 8, we considered the evidence for reconceptualisation as *partial and patchy* because the language consistent with reconceptualisation was accompanied by the language that was consistent with a biomedical understanding of pain. For example, in her interview before PNE, P1's response to being asked about the cause of her back pain was

*“Sclerosis…I know I've got disc degeneration.”* (P1 pre)

After PNE, she introduced neurophysiological language using the phrase “new nerve” in relation to neuroplasticity.

*“…it is the new nerve in sending the messages up…”* (P1 post)

while still describing the current cause of her pain in structural terms as before PNE.

*“I know I've got sclerosis of my lower back…whether the arthritis is starting to affect it more I don't know.”* (P1 post)

Participant 4, however, showed strong signs of reconceptualisation that exceeded partial reconceptualisation. He demonstrated the clearest change from pre- to post-PNE with respect to his explanation of his pain and his appreciation of the role of psychosocial factors on his pain. Both showed a clear shift away from the medical model. Prior to PNE, the participant believed that the most likely cause of his back pain was a fracture that had shown up in an MRI scan based on consultations with two different health care professionals.

*“He showed me on the thing (MRI scan) with his finger, that looks like a stress fracture to your back.”* (P4 pre)

*“He (the health care professional) said, and he believed that I've probably like fractured a couple of bones in my body.”* (P4 pre)

After PNE, P4's explanation of his current pain was uniformly expressed in neurophysiological language with an absence of the biomedical language that had dominated the interview before PNE.

*“…any slight jarring, or anything like that, and it sends my back into spasm, which is like just basically creating a protective shell and it's so used to doing it it's on hypersensitive and I think that's generally why my pain is, and it's just not switching off…(Interviewer: What causes that hypersensitivity?) …I think that's all those too much chemicals in my body.”* (P4 post)

Also, he showed a clear change in understanding of the link between pain and mood from tenuous

*“…I won't completely reject it…”* (P4 pre)

to a full acceptance of the links.

*“…the psychology…and stuff like that is massive and knowing how your brain works and stuff like that is huge…”* (P4 post)

Participant 14 was a unique case. With a university-level educational background in biology, P14 had developed a clear understanding of pain mechanisms consistent with reconceptualisation as seen in his interview before PNE.

*“…I've had possibly a few back problems…and my back has picked up on this, if you like the nerve has picked up on this, it's sent the signals to the brain, the brain's sent it back down and it probably happens over two or three months.”* (P14 pre)

That understanding did not change after PNE but was reinforced.

### 3.2. Theme 2: Personal Relevance

Even though he already had a clear understanding of pain mechanisms, P14 did find the session relevant to his own condition.

*“it all it did was to completely reaffirm the way that I was actually going or the way I'd actually thought before I came but you did it did help to if you like allay any I was going to say fears but it's not so much fears it's more concerns that I had in many ways, I'm going round the twist.”* (P14 post)

Of the 7 participants in whose accounts we observed evidence of reconceptualisation, we counted 5 as having applied that reconceptualisation to their own particular circumstances—P1, 2, 3, 4, and 7. In other words, their new understanding had personal relevance. Typically, this was noted by clear use of the first person singular such as

*“…basically the cause of my pain, my pain is sort of constant…”* (P4 post)

and by clear statements discussing the relevance of the session.

*“…at the time things that she was explaining did make sense and how, you know, things just triggered and how it all moves around your body and your mind and everything…I could relate to it, I could relate to it.”* (P7 post)

In contrast P8's account of reconceptualisation was more theoretical and related to a more general experience of living with pain, and when he described his own condition, the language was explicitly biomedical. Participants 9, 11, and 12 showed no clear evidence of relevance and indeed Participant 11 made it clear that she saw PNE as just another of the many things she was open to trying to help with her pain.

*“If you offered another session to me I'd still go, whether it was 100% relevant to me or not, I'll take anything that's going, I won't knock anything.”* (P11 post)

Participant 12 also reported a lack of relevance. His problems were pain and numbness in his legs following back surgery that had reduced pain in his back and he lamented the lack of a particular focus on his personal circumstances in the session.

*“…I didn't get the chance to explain what my problems were…it was about pain in general but it wasn't targeted at myself or anybody specific, it was just like everybody.”* (P12 post)

### 3.3. Theme 3: Importance of Prior Beliefs

Before PNE, all three participants in whom we found no reconceptualisation (P9, 11, and 12) believed that their current pain was caused by biomechanical factors and did not show any signs of dissatisfaction with this belief. The beliefs of Participants 9 and 12 were passive in that they had not really given other potential causative factors consideration while Participant 11 was actively opposed to any alternative explanation—indeed, she had walked out of a previous consultation when the clinician enquired about social issues.

*“…all she wanted to know about was my personal life and I walked out because I said I'm not here about anything other than a crash…”* (P11 pre)

Participant 8, whose reconceptualisation was general rather than personal, had a steadfast belief that his current pain was caused by damage to his facet joints. For the other six participants in whom we did find some reconceptualisation and relevance (P1, 2, 3, 4, 7, and 10), all apart from Participant 1 stated prior beliefs which demonstrated either a dissatisfaction with their existing biomedical explanation of the current pain.

*“…the only thing I've been told as well it's probably mechanical…I'm not convinced that it is mechanical, it's not the same kind of pain as on the left side…”* (P3 pre)

and/or an openness to a more biopsychosocial/neurophysiological sensitisation explanation consistent with PNE.

*“I think I've got a lot of nerve, I know I've got a lot nerve damage…I think it's just those nerve endings suddenly coming alive again…I presume it's just that message going to my brain saying you're in pain, that's all I'm thinking you know, I don't know if that's correct.”* (P7 pre)

### 3.4. Theme 4: Perceived Benefit of PNE

Neither Participant 8 nor Participant 10 described any clinical benefit from their PNE session. In the case of P8, rather than showing a clinical benefit after PNE, he discussed scenarios that were at odds with the aims of PNE. Most marked were statements about restricting movement and activity because of the potential damage to structures in his back.

While he offered an explanation for back pain linked to neurophysiology following PNE,

“… a build-up of the gateways being open permanently…allowing sensation to override…”

he clearly continued to link his pain with tissue damage.

*“I think it's telling me be careful…because you don't want to aggravate an injury or a potential injury or something's going to happen if you continue with that activity.”* (P8 post)

The context of this was that he was comfortable with the facet joint diagnosis that he had received and its plausibility was enhanced because he had experienced benefit from a stretching regime that he could rationalise in terms of that diagnosis. That ties in with his general rather than personal reconceptualisation.

P14 reported clinical benefit mainly in terms of reinforcement of his current understanding

*“…all it did was to completely reaffirm the way that I was actually going or the way that I'd actually thought before I came to you…”* (P14 post)

and clarification of some concerns that were causing him confusion.

*“…it did help to, if you like, allay any, I was going to say fears, but it's not so much fears, it's more concerns that I had in many ways, I'm going round the twist.”* (P14 post)

The remaining participants who we considered to have showed various degrees of partial reconceptualisation (P1, 2, 3, 4, and 7) all spoke about benefits from PNE. These described improved understanding about their pain and its management;

*“It made a lot of sense as to why even though especially over the last three or four years and all they've been doing is upping the painkillers why I'm not getting the relief that I thought I would be getting off them.”* (P3 post)

an increased ability to cope with pain;

*“…I suppose it's the acceptance what I've got out from this session is like to trying to accept the fact that you've got the pain for life and it's how that pain is managed is what makes life more manageable in itself.”* (P2 post)

and functional improvements.

*“…when I was walking quite briskly I just slowed down. I thought, oh calm down you've got plenty of time to get there…where before I would have just carried on…”* (P7 post)

Here, P7 describes how her new understanding of her pain influenced her walking in a form of activity pacing to carry on functioning while still experiencing pain.

Those who did not show signs of reconceptualisation under our criteria (Participants 9, 11, and 12) showed neither personal relevance nor clinical benefit.

*“It was more interesting than useful.”* (P11 post)

Participant 2 provided the first example in the literature of evidence of an adverse effect from PNE in that she found the session to be upsetting. She explained how the PNE instructor had given an example of someone who injured his back falling off a ladder and then found his pain triggered when he saw a ladder. From that example, Participant 2 recognised how she associated her back pain with childbirth and that now the presence of her child was acting as a trigger for her pain.

*“They made a reference to a person who had chronic back pain after having fallen off a ladder and every time they saw a ladder or had to go anywhere near a ladder it triggered the pain, made it worse, and although that's nothing like my situation it made me worry because my back pain is related to childbirth that the effects my pain was having on my family… I was upset to think that my pain was sometimes worse when my daughter was being more demanding and although that scenario that was given that person could spend a good quality of their life avoiding the situation, avoiding using a ladder, avoiding going near a ladder, I don't want to and couldn't even if I did want to avoid the situation of being a parent…I mean it was just that the pain could be associated to the cause and knowing the cause of my pain was my daughter initially though it wasn't her fault.”* (P2 post)

## 4. Discussion

This study aimed to explore the extent, and nature, of patients' reconceptualisation of their CLBP following PNE. The study investigated if the findings from our previous studies on reconceptualisation with PNE for people with chronic pain were sufficient to describe the experience of people specifically with CLBP. We found that the a priori themes—degrees of reconceptualisation, personal relevance, importance of prior beliefs, and perceived benefit of PNE—were all clearly identifiable within the data and did indeed provide a good description of participants' accounts.

Our finding of partial and patchy reconceptualisation, whereby participants showed a range of degrees of reconceptualisation including none, is similar to what we found previously [23, 24]. Our earlier observation of the importance of prior beliefs applies here as well. This time, however, we found strong signs of reconceptualisation in one participant, P4. What was interesting was that his prior beliefs were not notably dissimilar to that of others.

The role of prior beliefs of participants within our study were in keeping with the four steps to accommodate a new scientific concept outlined by Posner et al. [25]: (1) dissatisfaction with current beliefs, (2) the new concept making sense to the person, (3) plausibility of the new concept, and (4) a belief that the new concept will be of practical help to the person. Broadly, those who showed no signs of reconceptualisation showed no signs of dissatisfaction with their existing biomedical explanation for their pain while the majority of those who did show signs of reconceptualisation were open to the neural sensitisation explanation of pain within PNE as plausible/relevant/potentially helpful.

P4 shows that it is possible to achieve advanced reconceptualisation after one session. However, for most, it seems that more sessions would be required. P14's report of clinical benefit further highlights the importance of the availability of follow-up education. This was someone who had already acquired a high level of reconceptualisation and was functioning at a high level but was suitably troubled to seek help from a pain clinic. His expressed need was clarification of some issues that were causing him problems.

Another finding in this study that we had not come across before was the distress experienced by P2. She reported the distress as happening during the session and it was evident at the time of the interview three weeks after. We do not have any insight into how long if at all the distress continued into the longer term. This is the first reporting of an adverse event associated with PNE in the literature. The participant was offered the opportunity to discuss their feelings with a clinical psychologist; however, they did not think this was necessary and therefore declined the offer.

The lack of long-term follow-up is a limitation of this study. Pain management is an ongoing process and this is an important gap in knowledge. As highlighted by the needs expressed by P14 for education despite having a long history of managing his pain successfully, it would be foolish to think that people would never need further education and advice. The lack of data saturation could also be viewed as a limitation of this study [34]. However, this study did not attempt to achieve data saturation. The need for data saturation in all qualitative studies has not been established and it has been proposed that using saturation as a generic marker of qualitative research quality is misplaced [35]. The sample size employed in this study is in keeping with previous recommendations for studies which aim to *understand common perceptions and experiences* within a homogenous group [30].

As we have previously demonstrated [23, 24], relevance was once again seen as catalytic in the clinical impact of PNE. Interestingly, in Participant 8, we found an example of a participant who had misinterpreted the information to reinforce their maladaptive beliefs and behaviour having come across this in one of our previous studies [24]. This may reflect a form of confirmation bias that has been noted in the learning of scientific concepts [36]. Again, this reinforces the need for follow-up education and support.

A strength of the study was the use of interviews before and after the PNE session, which allows greater insight into changes in beliefs than would be obtained by only interviewing people after PNE. The coherence of the themes between our previous work and the current findings lends confidence to the certainty of this evidence [37]. That said, at this stage, the findings are still subject to the limitations of qualitative research as outlined in our last study [24] with the findings being illustrative rather than representative with limitations determined by the delivery of PNE by way of a single session, the close proximity between the post-PNE interviews and the delivery of the session, and the restriction of the sample to people whose first language is English.

### 4.1. Recommendations for Future Research

Important further work is needed to develop a method, probably using a questionnaire, to allow quantification of reconceptualisation so that a statistical approach can be used to produce more representative findings. This would require careful preliminary work to develop such a questionnaire with appropriate validity and reliability of a potentially mercurial construct. A useful starting point could be the pain neurophysiology quiz which has been developed and revised as a method of assessing change in knowledge of pain physiology information [38]. Also, further work is required to extend the qualitative approach used here to explore the delivery issues stated above.

Given the importance of the personal relevance of the information provided to the patient in PNE identified in this study and our previous work [23, 24], PNE may be most effective when the information is tailored to the individual. This would be in keeping with Moseley who found that PNE was clinically more effective, though less cost-effective, when delivered in a one-to-one compared to a group setting [19]. Future work should explore if PNE delivered in a homogenous patient group setting (e.g., a group of patients with CLBP) facilitating a more tailored group approach would maximise both clinical and cost-effectiveness. Patient group-specific PNE curricula are already available for a range of specific pain groups including people with CLBP [39]. Another clinical approach to facilitate tailoring of the material, to enhance relevance, could be to have the educating therapist undertake a thorough examination of the patient prior to delivering PNE. The examination could be used as a way of identifying individual patient issues (e.g., anxieties, fears, and misconceptions) that could be specifically targeted during the education session. Again, future work should explore if this would enhance the effectiveness of PNE.

PNE may be most effective when delivered in combination with other interventions, such as exercise, compared with when it is delivered in isolation [8, 10] as in this study. It would be interesting to explore qualitatively the extent and nature of patients' pain reconceptualisation following PNE delivered as part of a comprehensive multimodal package of care. Finally, health care professional's beliefs about pain can influence their clinical management of their patients. PNE has been shown to enhance health care students' understanding of pain and increase their likelihood of making appropriate recommendations for patients in practice. [40, 41]. However, that work has been of a quantitative nature and there is a need to further explore health care professional student's experience of PNE and the extent and nature of their pain reconceptualisation qualitatively.

## 5. Conclusion

This study aimed to explore the extent, and nature, of patients' reconceptualisation of their CLBP following PNE using a set of a priori themes developed from previous research with heterogeneous samples of pain patients. We found that patients with CLBP who received PNE underwent varying levels of reconceptualisation, that the degree of reconceptualisation was influenced by previous beliefs and how relevant the information was deemed by the patient. Furthermore, the degree of reconceptualisation appeared to be related to the perceived benefit reported by the patient. No new themes beyond the a priori themes emerged. The findings were in keeping with our previous work, which included chronic pain participants from a range of clinical groups including multisite pain, back pain, and complex regional pain syndrome. The applicability of the four a priori themes, developed in previous heterogeneous pain samples, indicates that the key experiences of PNE for those with back pain are similar to those identified within samples of patients consisting of heterogeneous pain groups.

## Figures and Tables

**Table 1:** Participant demographics and thematic analysis for each of the four a priori themes.

Id	Age (yrs)	Sex	Duration of pain (yrs)	Work status	Pre		Post			
					Belief that pain may not be due to tissue damage	Awareness of an emotion-pain relationship	Tissue damage reconceptualisation	Role of emotion reconceptualisation	Personal relevance	Perceived benefit
P1	42	F	22.0	Unemployed	No	No	Partial	Yes	Yes	Yes
P2	51	M	26.0	Unemployed	No	Partial	Partial	No	Yes	Yes
P3	44	F	6.0	Employed	No	Yes	Partial	Partial	Yes	Yes
P4	29	M	3.0	Employed	Yes	Yes	Yes	Yes	Yes	Yes
P6	25	F	4.5	Employed						
P7	46	F	10.0	Unemployed	Yes	Yes	Partial	Yes	Yes	Yes
P8	55	M	8.0	Retired	No	Partial	Partial	No	No	No
P9	72	F	5.0	Retired	No	Yes	No	No	Unclear	No
P10	40	F	22 .0	Employed	No	No	Partial	No	Unclear	—
P11	62	F	0.7	Retired	No	Partial	No	No	No	No
P12	56	M	7.0	Employed	No	No	No	No	No	—
P14	58	M	3.0	Employed	Yes	Partial	Yes	—	Yes	Yes

Participant's prior beliefs, degree of reconceptualisation, perceived relevance of PNE, and perceptions of benefit are shown. The tissue damage reconceptualisation and role of emotion reconceptualisation categories looked at change from pre-PNE. Blank (—) spaces indicate that the issue was not discussed. “Yes” and “No” are used when there was clear evidence related to the theme and partial when there was tentative evidence. Unclear is used when the issue was discussed, but it could not be determined whether the evidence supported or refuted the issue. P6 did not provide a second interview. F = females, M = male.

## Data Availability

The data used to support the findings of this study are available from the corresponding author upon request.

## Supplementary Materials

supplementary material Supplementary Materials The supplementary material contains the semistructured interview script.

## References

[1] Moseley G. L., Butler D. S. (2003). *Explain Pain*.

[2] Moseley G. L. (2002). Combining physiotherapy and education is efficacious for chronic low back pain. *Australian Journal of Physiotherapy*.

[3] Moseley G. L. (2003). Unravelling the barriers to reconceptualization of the problem in chronic pain: the actual and perceived ability of patients and health professionals to understand the neurophysiology. *Journal of Pain*.

[4] Moseley G. L., Nicholas M., Hodges P. (2004). A randomised controlled trial of intensive neurophysiology education in chronic low back pain. *Clinical Journal of Pain*.

[5] Ryan C. G., Gray H. G., Newton M., Granat M. H. (2010). Pain biology education and exercise classes compared to pain biology education alone for individuals with chronic low back pain: a pilot randomised controlled trial. *Manual Therapy*.

[6] Clarke C. L., Ryan C. G., Martin D. J. (2011). Pain neurophysiology education for the management of individuals with chronic low back pain: a systematic review and meta-analysis. *Manual Therapy*.

[7] Van Oosterwijck J., Meeus M., Paul L. (2013). Pain physiology education improves health status and endogenous pain inhibition in fibromyalgia: a double-blind randomized controlled trial. *Clinical Journal of Pain*.

[8] Moseley G. L., Butler D. S. (2015). 15 years of explaining pain–the past, present and future. *Journal of Pain*.

[9] Geneen L. J., Martin D. J., Adams N. (2015). Effects of education to facilitate knowledge about chronic pain for adults: a systematic review with meta-analysis. *Systematic Reviews*.

[10] Louw A., Zimney K., Puentedura E. J., Diener I. (2016). The efficacy of pain neuroscience education on musculoskeletal pain: a systematic review of the literature. *Physiotherapy Theory and Practice*.

[11] Darlow B., Fullen M., Dean S., Hurley D. A., Baxter G. D., Dowell A. (2012). The association between health care professional attitudes and beliefs and the attitudes and beliefs, clinical management, and outcomes of patients with low back pain: a systematic review. *European Journal of Pain*.

[12] Darlow B., Dowell A., Baxter G. D., Mathieson F., Perry M., Dean S. (2013). The enduring impact of what clinicians say to people with low back pain. *Annals of Family Medicine*.

[13] Bunzli S., Watkins R., Smith A., Schütze R., O’Sullivan P. (2013). Lives on hold: a qualitative synthesis exploring the experience of chronic low-back pain. *Clinical Journal of Pain*.

[14] Lin I. B., O’Sullivan P. B., Coffin J. A., Mak D. B., Toussaint S., Straker L. M. (2013). Disabling chronic low back pain as an iatrogenic disorder: a qualitative study in Aboriginal Australians. *BMJ Open*.

[15] Leeuw M., Goossens M. E., Linton S. J., Crombez G., Boersma K., Vlaeyen J. W. (2007). The fear-avoidance model of musculoskeletal pain: current state of scientific evidence. *Journal of Behavioral Medicine*.

[16] Eccleston C., Crombez G. (2007). Worry and chronic pain: a misdirected problem solving model. *Pain*.

[17] Moseley G. L. (2007). Reconceptualising pain according to modern pain science. *Physical Therapy Reviews*.

[18] Fletcher C., Bradnam L., Barr C. (2016). The relationship between knowledge of pain neurophysiology and fear avoidance in people with chronic pain: a point in time, observational study. *Physiotherapy Theory and Practice*.

[19] Moseley G. L. (2003). Joining forces-combining cognition-targeted motor control training with group or individual pain physiology education: a successful treatment for chronic lower back pain. *Journal of Manual and Manipulative Therapy*.

[20] Moseley G. L. (2004). Evidence for a direct relationship between cognitive and physical change during an education intervention in people with chronic low back pain. *European Journal of Pain*.

[21] Magilvy J. K., Thomas E. (2009). A first qualitative project. Qualitative descriptive design for novice researchers. *Journal for Specialists in Pediatric Nursing*.

[22] Pope C., Mays N. (1995). Researching the parts other methods cannot reach: an introduction to qualitative methods in health and health services research. *BMJ*.

[23] Robinson V., King R., Ryan C. G., Martin D. J. (2016). A qualitative exploration of people’s experiences of pain neurophysiological education for chronic pain: the importance of relevance for the individual. *Manual Therapy*.

[24] King R., Robinson V., Ryan C. G., Martin D. J. (2016). An exploration of the extent and nature of reconceptualisation of pain following pain neurophysiology education: a qualitative study of experiences of people with chronic musculoskeletal pain. *Patient Education and Counseling*.

[25] Posner J. G., Strike K. A., Hewson P. W., Gertzog W. A. (1982). Accommodation of a scientific conception: toward a theory of conceptual change. *Science Education*.

[26] O’Sullivan P. (2012). It’s time for change with the management of non-specific chronic low back pain. *British Journal of Sports Medicine*.

[27] Murray C. J., Richards M. A., Newton J. N. (2013). UK health performance: findings of the Global Burden of Disease Study 2010. *The Lancet*.

[28] NICE *Low Back Pain: The Early Management of Persistent non-Specific Low Back Pain. NICE Clinical Guideline 88*.

[29] Braun V., Clarke V. (2006). Using thematic analysis in psychology. *Qualitative Research in Psychology*.

[30] Guest G., Bunce A., Johnson L. (2006). How many interviews are enough? An experiment with data saturation and variability. *Field Methods*.

[31] Robinson V., King R. (2012). “Explain pain” as part of a pain management service improves patient’s understanding of the neurophysiology of chronic pain. *Pain and Rehabilitation-The Journal of Physiotherapy Pain Association*.

[32] Robinson V., King R., Ryan C. G. (2013). Pain neurophysiology education” as part of a pain management service decreases fear avoidance and improves patient’s understanding of the neurophysiology of chronic pain at four months follow up. *Pain and Rehabilitation-The Journal of Physiotherapy Pain Association*.

[33] Jootun D., McGhee G., Marland G. R. (2009). Reflexivity: promoting rigour in qualitative research. *Nurs Stand*.

[34] Morse J. M. (1995). The significance of saturation. *Qualitative Health Research*.

[35] O’Reilly M., Parker N. (2013). “Unsatisfactory saturation”: a critical exploration of the notion of saturated sample sizes in qualitative research. *Qualitative Research*.

[36] Chinn C. A., Brewer W. F. (1993). The role of anomalous data in knowledge acquisition: A theoretical framework and implications for science instruction. *Review of Educational Research*.

[37] Lewin S., Glenton C., Munthe-Kaas H. (2015). Using qualitative evidence in decision making for health and social interventions: an approach to assess confidence in findings from qualitative evidence syntheses (GRADE-CERQual). *PLoS Medicine*.

[38] Catley M. J., O’Connell N. E., Moseley G. L. (2013). How good is the neurophysiology of pain questionnaire? A rasch analysis of psychometric properties. *Journal of Pain*.

[39] Moseley G. L., Butler D. S. (2017). *Explain Pain Supercharged*.

[40] Colleary G., O’Sullivan K., Griffin D., Ryan C. G., Martin D. J. (2017). Effect of pain neurophysiology education on physiotherapy students’ understanding of chronic pain, clinical recommendations and attitudes towards people with chronic pain: a randomised controlled trial. *Physiotherapy*.

[41] Maguire N., Chesterton P., Ryan C. G. (2018). The effect of pain neurophysiology education on sports therapy and rehabilitation students’ knowledge, attitudes and clinical recommendations towards athletes with chronic pain. *Journal of Sport Rehabilitation*.

