# Supplementary material for: Pain Reconceptualisation after Pain Neurophysiology Education in Adults with Chronic Low Back Pain: A Qualitative Study
Source: Pain Res Manag. 2018 Sep 12;2018:3745651. doi: 10.1155/2018/3745651 (PMC6157134; doi:10.1155/2018/3745651)
Supplement: supplementary material Supplementary Materials — The supplementary material contains the semistructured interview script. [file 3745651.f1.docx]

**Supplementary material 1**

Interview Schedule.

Introduction.

Warm up questions.

1. Can you tell me about your pain?

2. How long have you had CP?

3. How did it start?

A. Current pain conception (Does pain represent tissue damage in the participants’ belief system?)

1. What is the cause of your pain?

a. If you were to tell a friend what causes your pain what you say?

b. Why does that cause pain?

2. What does you pain tell you about the state of your tissues?

a. Because you are in pain are your tissues injured.

3. How do you know that is the cause of your pain?

a. Without mentioning names, who told you/diagnosed your problem? (was it a Doctor/physiotherapist/other?)

b. Have you had other opinions?

c. Have you done your own research?

4. What evidence do you have for your understanding for the causes i.e. scans, test doctors.

B. Other contributing factors (pain can be modulated by many factors)

1. What affects your CP?

Prompts. Is your pain always the same level on a VAS or does it alter? If so, what alters it?

2. What makes it better?

3. What makes it worse?

Prompts. Physical/Emotional /Social factors

C. Length of time you have been in pain. (The longer pain persists the less predictable and weaker the correlation between pain and tissue damage become)

1. What does the length of time you have been in pain say about your pain?

Prompt. Has the problem heeled/not healed? Does it mean the problem has got worse or getting worse?

2. Does your pain behave the same way as it has always behaved?

3. Can you predict what will cause your pain?

4. Can you predict the way your pain will behave?

a. and have you always/ever been able to predict this?

Prompt. Activity/the weather hot cold/stress/depression/fatigue

D. Does worrying about the cause of pain correlate to pain levels

1. Do you associate your pain with danger to the tissues?

2. Are you worried about the cause of your pain?

3. Has the state of worry, (ether not worried or worried) changed over time?

4. In that time has your pain changed?

Prompt. Does the amount you worry about your pain effect the level of pain you are in?

**E. Second interview: Questions.**

Introduction

1. What did you think of the education session?

2. Was it relevant to you?

3. Was there any information that you had not heard before?

4. Did you understand the session?

5. Has it changed the way you understand your pain

Prompt.

6. Were you able to identify with any new contributing factors, for example, thing that might make your pain worse or better?

7. What are the cause of your pain? Are they the same now as before the session?

a. You said that your pain was due to your…. for example Back/neck/FMA

8. Has the session change any worries you might have had about your pain?

a. For example you said…. for example, you were worried about the stat of your back/future/wheelchair etc

9. If you have adopted a new belief about the cause or influences of/on your pain how relevant are they

a. How must is due to sensitisation how much is due to MSK origin?

10. Were there parts of the talk that you found more useful/relevant?

a. If so which part and why?

11. Rounding off

Is there any ways I which it could be improved?

Is there anything you would like to ask?
